# Case report: Applicability of breastfeeding the child of a patient with kidney failure with replacement therapy

**DOI:** 10.3389/fmed.2023.1098324

**Published:** 2023-02-10

**Authors:** Elena V. Kondakova, Anastasia E. Filat’eva, Nadezhda A. Lobanova, Egor I. Nagaev, Ruslan M. Sarimov, Sergey V. Gudkov, Maria V. Vedunova

**Affiliations:** ^1^Institute of Biology and Biomedicine, Lobachevsky State University of Nizhny Novgorod, Nizhny Novgorod, Russia; ^2^Branch FESFARM NN, Nizhny Novgorod, Russia; ^3^Prokhorov General Physics Institute of the Russian Academy of Sciences, Moscow, Russia

**Keywords:** case report, breastfeeding, immunology, Kidney Failure with Replacement Therapy (KFRT), biochemistry, molecular physics

## Abstract

This case report highlights the benefit or harm of breastfeeding in a patient with Kidney Failure with Replacement Therapy (KFRT) undergoing program hemodialysis. This is a unique clinical case, as pregnancy and successful delivery are rare in this group of females. With a favorable outcome, the possibility of breastfeeding is especially relevant for doctors and the mother. The patient was a 31-year-old female who was diagnosed in 2017 with end-stage renal disease associated with chronic glomerulonephritis. Against the background of hemodialysis, pregnancy, accompanied by polyhydramnios, anemia, and secondary arterial hypertension, occurred in 2021. At 37 weeks, a healthy, full-term baby girl was born, and breastfeeding was started. In this study, we conducted a detailed analysis of toxic substances and immunologically significant proteins using high-tech analysis methods. In addition, we studied different portions of milk before and after hemodialysis at different time intervals. After a wide range of experiments, our study did not reveal an optimal time interval for breastfeeding a baby. Despite the decrease in the level of the major uremic toxins 4 h after the hemodialysis procedure, their level remained high. In addition, the content of nutrients did not reach acceptable limits and the immune status was characterized as pro-inflammatory. In our opinion, breastfeeding is not advisable for this group of patients since the concentration of nutrients is low, and the content of toxic substances exceeds the permissible limits. In this clinical case, the patient decided to stop breastfeeding one month after delivery due to insufficient breast milk and the inability to express it in a certain period of time.

## Introduction

Motherhood with Kidney Failure with Replacement Therapy (KFRT) requires critical and comprehensive attention, starting with the possibility of pregnancy, its maintenance at high risks of miscarriage, to postpartum complications. Patients with KFRT are approximately 100 times less likely to have a live birth (1) than a female without kidney disease. Nevertheless, thanks to the advances in medicine and the well-coordinated work of a council of physicians, the number of successful pregnancies in patients on hemodialysis has been growing in recent years.

From an evolutionary and nutritional perspective, exclusive breastfeeding during the first 6 months of life has been recognized as the gold standard of infant nutrition (2). The benefits and indispensability of breastfeeding for the harmonious development of the child have been confirmed by numerous studies (3–5). Milk promotes optimal growth of the child, including the establishment of circadian rhythms, the production of protective antibodies, and the formation of a healthy gut microbiome (6).

However, the benefits of milk have been well demonstrated for healthy mothers; little is known about how KFRT and hemodialysis procedures affect the quality and benefits of breast milk or whether maternal toxins and metabolites pass from the blood into milk.

Elevated levels of uremic toxins impair the normal functioning of various organ systems. In particular, the neurotoxic effect of water-soluble guanidine compounds, which include creatinine, was shown (7). In addition, uremic toxins disrupt endothelial functions causing structural damage, inflammation, and disruption of endothelium-dependent vasodilation (8, 9).

Uremic toxins affect both the innate and adaptive immune systems through multiple mechanisms, leading to systemic pathologies in humans. The accumulation of uremic molecules and cytokines activates the innate immune response, leading to a vicious cycle that stimulates the production of cytokines and ROS, which are known to increase the risk of cardiovascular disease and tissue damage (10). Moreover, uremic toxins have a pro-apoptotic and/or inhibitory effect on immune cells, contributing to an increased risk of infections (11, 12).

Uremic toxins can lead to diarrhea by disrupting the intestinal microflora. Hydrolysis of urea with the formation of a large amount of ammonia increases the intestinal pH, which leads to irritation of the intestinal mucosa and may have a negative effect on the growth of commensal bacteria that contribute to the maintenance of intestinal dysbacteriosis (13).

All of the above raises the question of the potential harm of breast milk, including it in the long term. Therefore, neonatologists and pediatricians face the important question of whether breastfeeding should be recommended to mothers on hemodialysis.

## Case presentation

The patient was a 31-year-old female diagnosed in 2017 with end-stage renal disease associated with chronic glomerulonephritis. Family, genetic, and psychosocial history was not aggravated. She underwent a living-related kidney transplantation, but the transplant was removed due to chronic cellular humoral rejection. In 2021, she was transferred to renal replacement therapy using the program hemodialysis. In the same year, against the background of hemodialysis, she had a pregnancy accompanied by polyhydramnios, anemia, and secondary arterial hypertension. This was the second pregnancy of the female: the first (before KFRT) ended in a spontaneous miscarriage in the second trimester. The patient was offered a therapeutic abortion, but it was refused. During pregnancy, the regimen of renal replacement therapy was changed: the program hemodialysis procedure was carried out for 3 h 30 min 6 times a week. Dialysis regimen: hemodiafiltration; weekly dialysis time—20–24 h; vascular access—native arteriovenous fistula; blood flow rate—320 ml/min; the dialysate flow rate—500 ml/min; Fresenius FX 60 dialyzer. Hemostabilization during the procedure—enoxaparin sodium. To correct anemia, the dose of erythropoietin was increased from 4,000 to 6,000 IU/week. Iron (III) hydroxide sucrose complex dose increased from 100 to 200 mg per month (intravenously).

Biochemical and ultrasound screening at 19 weeks revealed congenital malformations in the fetus (pyelectasis and megaureter on both sides). At the 23rd week, hemostatic therapy was performed in labor due to the threat of premature delivery. A diet with sodium, phosphorus, and potassium restrictions, compliance with the water-drinking regime was recommended. Antihypertensive therapy: Methyldopa 250 mg × 2–3 times a day while controlling blood pressure. Iodine, folic acid, ascorbic acid, calcium preparations, vitamin D were prescribed. Therapy with gestagens (Utrogestan 200 mg × once a day) was carried up to the 34th week. At the 28th week, isthmic-cervical insufficiency was corrected with an obstetric pessary. Antibiotic therapy was conducted to prevent infectious complications (Cefazolini 1.0).

At the 37th week, a planned cesarean section was performed for delivery. A lower midline incision was performed in order to prevent trauma to the iliac regions for reinsertion of the renal graft. A live full-term girl, weighing 2,460 g and 47 cm tall, was retrieved. Apgar score was 8/9 points. The dynamics of renal function parameters during pregnancy and after delivery are shown in Supplementary Table 1. The mother’s conscious decision was to start breastfeeding.

At the first stage of the study, we found that the composition of the breast milk of a patient on hemodialysis differed from that of healthy mothers in the control group in several aspects. Fresh portions of milk a month after birth were used to assess the level of milk’s biochemical parameters, including the major uremic toxins (creatinine, urea).

In our study, we used urea (60 Da) and creatinine (113 Da) as the most representative low molecular weight uremic toxins that are widely used as surrogate markers for detecting kidney function in the clinic. Urea and creatinine are easy to measure with reliable and inexpensive assays in routine laboratory practice (14).

Since there are no reference values for most of the analytes we measured in milk, the milk of healthy females (*n* = 5) with a similar duration of breastfeeding, collected with informed voluntary consent, served as a control. When studying the level of biochemical parameters in milk samples, a significant increase in the content of uremic toxins, particularly creatinine and urea, in milk both before hemodialysis and after the procedure compared with control samples of healthy females was shown.

**FIGURE 1:**
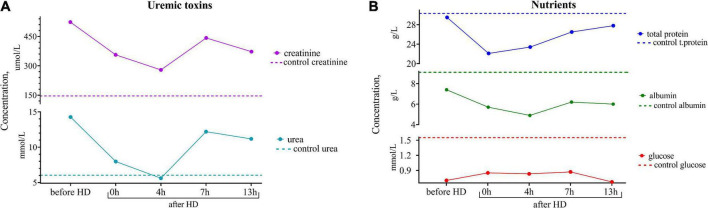
**(A)** Dynamics of uremic toxins (creatinine and urea) and **(B)** nutrients (total protein, albumin, and glucose) concentrations in the breast milk of the patient undergoing renal replacement therapy by program hemodialysis. The horizontal axis presents time relative to the hemodialysis procedure: before HD, immediately after HD (0 h), 4, 7, and 13 h after hemodialysis. The vertical axis shows the concentration of analytes. Dashed lines indicate the average concentration of each analyte in the control sample (breast milk of healthy females with the same lactation period).

Since the female was highly motivated to breastfeed, it was of the utmost importance for the clinicians to assess the effect of the hemodialysis procedure on her breast milk over time and to find the optimal time to express the least dangerous portions of milk. For this purpose, samples of the patient’s breast milk were collected for further research at different time intervals during the day: before the hemodialysis procedure; immediately after the hemodialysis; 4, 7, and 13 h after the end of the hemodialysis procedure. The experiments showed that the lowest content of creatinine and urea was observed not immediately after the hemodialysis procedure but 4 h after its completion, which could be explained by the redistribution of toxic substances from the tissues into the blood (Figure 1).

**FIGURE 2:**
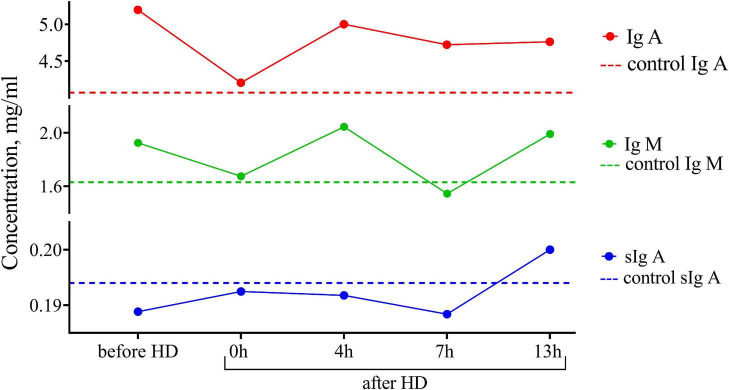
Dynamics of the concentration of immunoglobulins of IgA, IgM, and sIgA subclasses in the breast milk of the patient undergoing renal replacement therapy by program hemodialysis. The horizontal axis presents time relative to the hemodialysis procedure: before HD, immediately after HD (0 h), 4, 7, and 13 h after hemodialysis. The vertical axis shows the concentration of analytes. Dashed lines indicate the average concentration of each analyte in the control sample (breast milk of healthy females with the same lactation period).

One of the advantages of breastfeeding is the stimulating effect of breast milk on the humoral development of immunity, which leads to a decrease in the incidence of illness in children. In this regard, we assessed the concentration of immunoglobulin IgA, sIgA, and IgM subclasses in breast milk (Figure 2). The content of immunoglobulins did not differ from the control, and there were no significant peaks in the dynamics of concentrations before and after hemodialysis.

**FIGURE 3:**
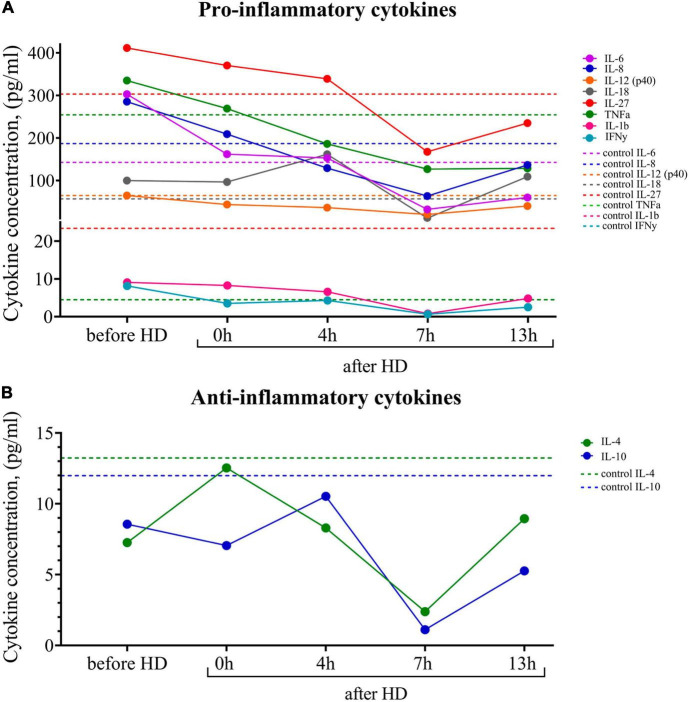
**(A)** Dynamics of pro-inflammatory and **(B)** anti-inflammatory cytokine concentrations in the breast milk of the patient undergoing renal replacement therapy by program hemodialysis. The horizontal axis presents time relative to the hemodialysis procedure: before HD, immediately after HD (0 h), 4, 7, and 13 h after hemodialysis. The vertical axis shows the concentration of analytes. Dashed lines indicate the average concentration of each analyte in the control sample (breast milk of healthy females with the same lactation period).

Breast milk is known to be the main source of cytokines, especially anti-inflammatory ones, for newborns, who are usually deficient in these proteins. Using multiplex cytokine analysis with the Luminex XMAP technology, we assessed a range of pro-inflammatory and anti-inflammatory cytokines in breast milk at different time points after hemodialysis. The results are presented in Figure 3. There was an increased level of pro-inflammatory cytokines in milk both before and after the hemodialysis procedure compared with the control. Pro-inflammatory cytokines, which, according to the literature data, also accumulate in uremia, belong to medium-molecular uremic toxins (15).

At the same time, there was a tendency for the level of pro-inflammatory cytokines to fall 7 h after the hemodialysis procedure. The level of the major anti-inflammatory cytokines (Il-4, Il-10) at all measurement points was lower than in the control samples.

**FIGURE 4:**
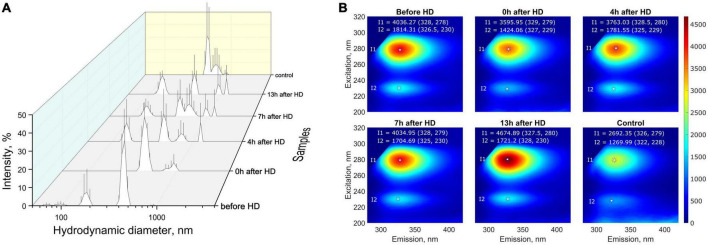
**(A)** Distribution (intensity weighted) of hydrodynamic particle diameters obtained by MADLS in milk samples from the patient with KFRT at various time intervals (before HD, immediately after HD (0 h), 4, 7, and 13 h after HD) and in control samples (milk of healthy females with the same lactation period); **(B)** fluorescence spectra of breast milk samples. Milk samples from a patient with KFRT have higher fluorescent activity compared with control samples.

For the most targeted study of milk composition, a series of experiments was carried out to investigate the molecular physical properties. Multi-angle dynamic light scattering (MADLS) was used to determine milk particle sizes, reflecting the diffusion properties of its constituent molecules. Based on the data on hydrodynamic diameter distributions, it can be concluded that low- (∼70–200 nm), medium- (∼400–500 nm), and high-molecular (500–1500 nm) compounds or aggregates were present in the milk samples of a patient with KFRT (Figure 4A). A study of milk samples using fluorescence spectroscopy showed that the patient’s milk sample collected before hemodialysis had a higher fluorescent activity compared to control samples (Figure 4B). The fluorescence intensity after hemodialysis decreased by 10%, approaching the fluorescence of the control sample. After that, the fluorescence increased, returning to its original value after 7 h. At the same time, the fluorescence emission peak shifted from 329 nm immediately after hemodialysis to 327.5 nm 13 h after the end of the procedure. The second fluorescence peak (327, 229 nm) also behaved unusually. After hemodialysis, the intensity of the second peak decreased by 20%, and after 4 h, it was restored to its original values before hemodialysis. A slight shift in the emission maximum during fluorescence from 326 to 328 nm may indicate a different protein composition of the milk of a healthy participant and a sick patient. This is also evidenced by the absorption of proteins in the UV (absorption due to amino acids) and the visible region (absorption due to aggregates and micelles), where the control sample absorbed more than the patient’s milk sample both before and after hemodialysis (the results are presented in Supplementary Table 1).

## Discussion

Pregnancy, childbirth, and breastfeeding in KFRT are courageous steps that only some patients dare to take. Breast milk is undoubtedly the best source of nutrition for an infant and has health benefits for the mother. It ensures the proper development of the immune system, programming of endocrine and metabolic functions (5). Breastfeeding is recommended whenever possible, with or without supplementary feeding, depending on the baby’s needs. However, the decision to breastfeed should be as balanced and deliberate as possible, taking into account the impact on the mother and child.

To the best of our knowledge, there are no clear guidelines for breastfeeding in chronic kidney disease (CKD) and KFRT. The only generally accepted recommendations are to counsel patients in the early stages of CKD and before kidney transplantation regarding pregnancy complications. A single case of breastfeeding in patients with CKD has been described in the literature. In their study, Balzer et al. (6) showed that breastfeeding may be a viable option for newborns from mothers on dialysis, as the composition of their breast milk has more similarities than differences with that of healthy mothers. The authors report that breastfeeding after a dialysis session is preferable to breastfeeding before dialysis. However, in this study, the conclusion about the applicability of breastfeeding is based primarily on immunoglobulin levels, which were found to be the same in healthy females and patients on hemodialysis.

We disagree with the statement above since the risk to the child of a mother with KFRT who is breastfed cannot be excluded. The decision to stop breastfeeding should be made based on an assessment of the benefits of breastfeeding for the baby and the benefits of therapy for the mother. Breastfeeding in KFRT is challenging, not only due to the impact of the hemodialysis procedure but also due to the adverse effects of drugs on infants and lactation itself. Experts also raise concerns about the mother’s ability to feed her baby, given her clinical condition after a complicated, high-risk pregnancy (4).

In our study, we conducted a detailed analysis of toxic substances and immunologically relevant proteins using high-tech analysis methods. The advantage of the approach is the study of milk portions before and after the hemodialysis procedure at different time intervals. As a result, after a wide range of experiments, our study did not reveal an optimal time interval for breastfeeding a child.

Despite the decrease in the level of major uremic toxins 4 hours after the hemodialysis procedure, their level remained high. It is known that high concentrations of urea can cause protein denaturation (16), which has an extremely bad effect on the composition of milk, blocks digestive enzymes, and changes the conformation of immunoglobulins (17). In addition, due to its osmotic properties, the urea solution can provoke diarrhea, which is very unfavorable for a baby in the first months of life.

Low and medium molecular weight compounds identified by MADLS are most likely associated with protein micelles (18), while high molecular weight compounds are associated with fat aggregates (19). However, such a division is rather arbitrary in the presence of lipoprotein micelles in a wide range of sizes (20). The presence of low molecular weight compounds that are not present in control samples may also be associated with the presence of toxic substances, such as certain lipoprotein modifications, advanced glycation end products (AGEs), and lipooxidation in KFRT patient milk, which are of comparable size (21). The changes in the fluorescence of patient’s samples after hemodialysis are of particular interest. These changes indicate that hemodialysis leads to significant changes in the protein content in the milk of a patient with KFRT.

Some experts believe that it is necessary to encourage and remove barriers to breastfeeding, where appropriate, for mothers with KFRT on dialysis or after transplantation. The authors refer to the fact that “breast milk is the perfect food.” However, it is important to remember that any nutritional deficiencies that exist during pregnancy will eventually be carried over through lactation (22). In our study, the content of nutrients, especially proteins, did not reach acceptable limits.

Since cytokines are known to activate and maintain the body’s immune response and reduce the risk of chronic diseases, we believe it is important to focus on their ratios in breast milk. Abnormal cytokine production may have negative health consequences and can contribute to the development of food allergies, jaundice, and immune disorders in later life (23–25). In the given case study, we note the pro-inflammatory status of the milk of a female with KFRT, which can negatively affect the child.

A limitation of the given study is the lack of clinical data on the condition of the child after birth.

## Conclusion

To sum up, the results obtained raise the question of the need for further research on the applicability of breastfeeding in patients with KFRT undergoing program hemodialysis. In our opinion, breastfeeding is not advisable for this group of patients since the concentration of nutrients is low, and the content of toxic substances exceeds the permissible limits. In this clinical case, the patient decided to stop breastfeeding a month after delivery due to insufficient breast milk and the inability to express it in a certain period of time.

## Data availability statement

The original contributions presented in this study are included in the article/Supplementary material, further inquiries can be directed to the corresponding author.

## Ethics statement

Written informed consent was obtained from the individual(s) for the publication of any potentially identifiable images or data included in this article.

## Author contributions

EK, SG, and MV contributed to conception and design of the study. EK and NL organized clinical data collection. EK and AF wrote the first draft of the manuscript. AF prepared the figures. EN, RM, and SG wrote sections of the manuscript. All authors contributed to manuscript revision, read, and approved the submitted version.
