# Supplementary material for: Case report: Applicability of breastfeeding the child of a patient with kidney failure with replacement therapy
Source: Front Med (Lausanne). 2023 Feb 10;10:1098324. doi: 10.3389/fmed.2023.1098324 (PMC9950765; doi:10.3389/fmed.2023.1098324)
Supplement: Supplementary file 1 [file Data_Sheet_1.docx]

Supplementary Material

**Supplementary Tables 1.** The dynamics of complete blood count and renal function parameters during pregnancy and after delivery

| **Dynamics of complete blood count** | | | | | | | | |
| --- | --- | --- | --- | --- | --- | --- | --- | --- |
| **Gestational age** | **12 weeks** | **23 weeks** | | **28 weeks** | **32 weeks** | **36 weeks** | **24 hours after delivery** | **1 month after delivery** |
| **Erythrocytes, 10^12^/L** | 3.33 | 4.16 | | 3.6 |  |  | 3.89 |  |
| **Hemoglobin, g/L** | 101 | 131 | | 120 | 114 | 126 | 127 | 104 |
| **Hematocrit, %** | 29 | 46.6 | | 36.6 | 34.4 | 37.6 | 39.8 | 32 |
| **Platelets, 10^9^/L** | 196 | 83 | | 120 | 119 | 133 | 107 | 150 |
| **Leukocytes, 10^9^/L** | 10.08 | 7.44 | | 8.10 | 8.1 | 7.56 | 10.88 | 5.59 |
| **Dynamics of indicators of kidney function** | | | | | | | | |
| **Gestational age** | | | **23 weeks** | **28 weeks** | **32 weeks** | **36 weeks** | **24 hours after delivery** | **1 month after delivery** |
| **Creatinine, µmol/L** | | | 573.6 | 437 |  | 393.3 | 607.6 | 656.9 |
| **Urea (before HD), mmol/L** | | | 17.54 | 7.1 | 14.21 | 8.11 | 15.24 | 14.77 |
| **Urea (after HD), µmol/L** | | |  |  | 3.81 | 2.31 |  | 4.13 |
| **% of decrease in urea** | | |  |  | 73 | 72 |  | 72 |
| **Kt/V** | | |  |  | 1.38 | 1.38 |  | 1.56 |
| **Calcium total, mmol/L** | | | 1.1 | 1.06 | 2.33 | 2.2 | 1.17 | 2.08 |
| **Phosphorus, mmol/L** | | |  |  | 0.71 | 0.69 |  | 1.13 |
| **Ca * P** | | |  |  | 1.65 | 1.5 |  | 2.35 |
| **ALP, unit/L** | | | 76.6 | 263 | 148.4 |  | 145.4 | 149.54 |
| **Albumin, g/L** | | | 31.6 | 35.3 |  | 34.08 | 28.9 |  |
| **ALT, unit/L** | | | 9.2 | 12 |  |  | 15.4 | 17.4 |
| **AST, unit/L** | | | 11.6 | 16 |  |  | 14.4 | 10.3 |
| **Cholesterol, mmol/L** | | | 4.53 | 6.0 | 5.33 |  | 4.81 |  |
| **Pre-dialysis blood pressure, mmHg** | | |  | 130/70 | 120/70 | 130/80 |  | 130/80 |
| **Post-dialysis blood pressure, mmHg** | | |  | 115/70 | 110/70 | 115/70 |  | 130/70 |

*
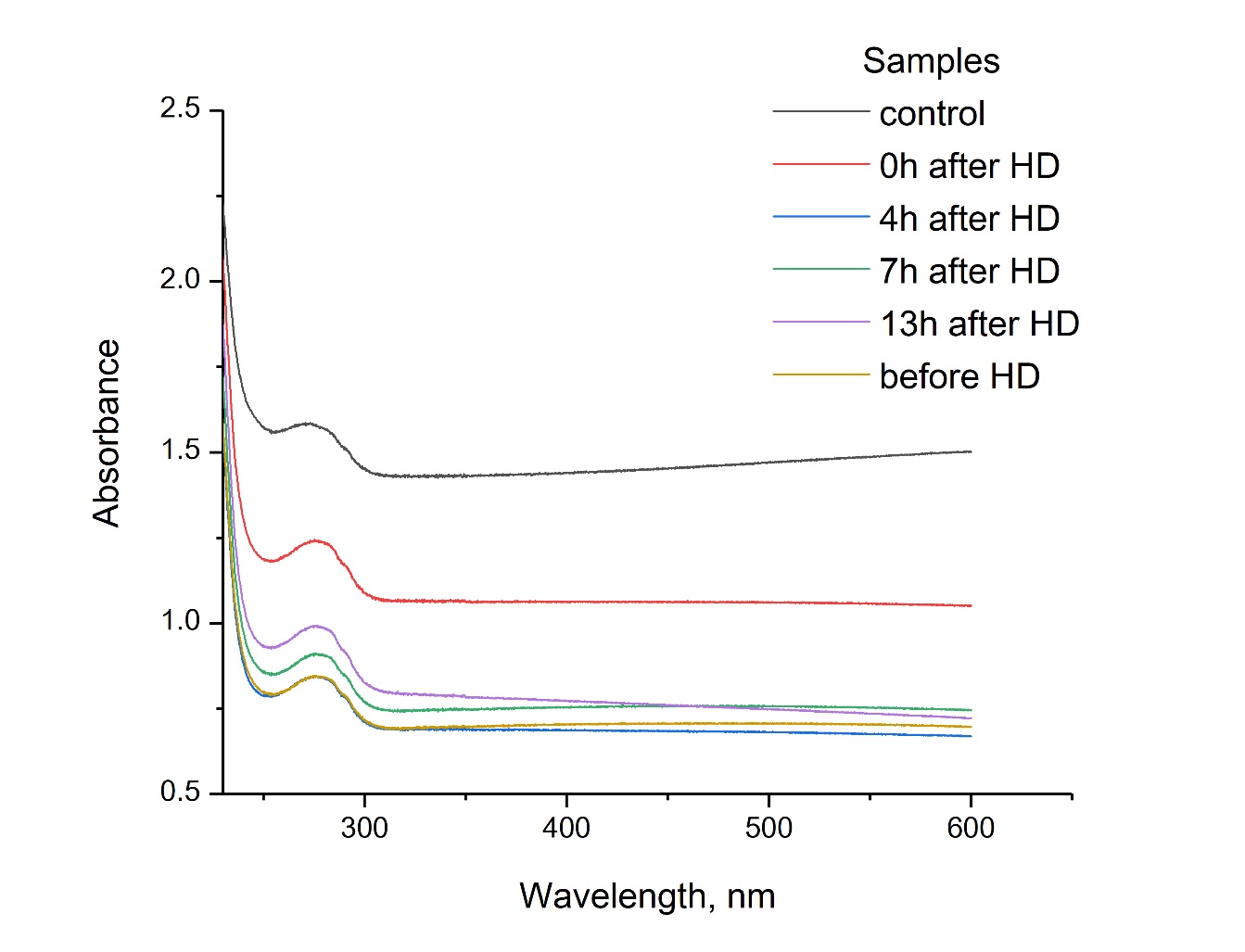
*

**Supplementary Figure 1.** Optical density for the control sample (healthy participant) and milk samples of the studied patient before hemodialysis and at various time intervals after hemodialysis.
